# Alpha-ketoglutarate promotes random-pattern skin flap survival by enhancing angiogenesis via PI3K/Akt/HIF-1α signaling pathway

**DOI:** 10.1186/s13619-025-00264-8

**Published:** 2025-12-22

**Authors:** Jiefeng Huang, Shuangmeng Jia, Yitong Ji, Yingjia Zhu, Yishu Lu, Yiming Tang, Jiajie Yang, Guangpeng Liu, Lei Cui, Shuaijun Li

**Affiliations:** 1 Department of Plastic Surgery, Shanghai Tenth Peoples Hospital, Tongji University School of Medicine, Shanghai, 200092 China; 2 Department of Stem Cells and Regenerative Medicine, Tongji University School of Medicine, Shanghai, 200072 China; 3 School & Hospital of Stomatology, Tongji University, Shanghai Engineering Research Center of Tooth Restoration and Regeneration, Shanghai, 200072 China; 4 Nantong Haimen Peoples Hospital, 226100 Nantong, China

**Keywords:** Glutamine metabolism, Alpha-ketoglutarate, Skin fap, Angiogenesis, PI3K/Aktpathway

## Abstract

Random-pattern skin flaps are widely employed in tissue reconstruction, however, their survival is frequently hindered by ischemia, leading to necrosis. Metabolic alterations have been implicated in playing critical roles in angiogenesis during tissue repair. Using RNA sequencing analysis in a mouse model, we identified significant disruptions in glutamine metabolism, which substantially impaired angiogenesis within random-pattern skin flaps. Although local glutamine repletion failed to alleviate ischemia, administering α-ketoglutarate (α-KG) markedly promoted angiogenesis, as evidenced at both gene and protein levels. In human umbilical vein endothelial cells,α-KG enhanced the stability of hypoxia-inducible factor (HIF-1) alpha through activation of the phosphoinositide 3-kinase (PI3K)-Akt signaling pathway. Notably, α-KG treatment improved flap viability by augmenting blood perfusion, an effect correlated with upregulation of vascular endothelial growth factor expression. Together, these results reveal a novel mechanism by which α-KG enhances random-pattern skin flap viability via promoting angiogenesis through the PI3K/Akt/HIF-1α pathway, offering promising therapeutic insights for improving flap survival.

**Supplementary Information:**

The online version contains supplementary material available at 10.1186/s13619-025-00264-8.

## Study highlights

1. Impaired glutamine metabolism and reduced α-KG levels are associated with necrosis in random-pattern skin flaps.
2. Local administration of α-KG significantly promotes survival and blood flow in ischemic skin flaps.
3. α-KG enhances angiogenesis by stabilising HIF-1α and upregulating VEGF in HUVECs.
4. The pro-angiogenic effect of α-KG is mediated through activation of the PI3K/Akt/HIF-1α signaling pathway.
5. Targeting glutamine metabolism represents a novel therapeutic strategy to improve skin flap survival by enhancing angiogenesis. **Keywords**: glutamine metabolism, alpha-ketoglutarate, skin fap, angiogenesis, PI3K/Aktpathway

**Supplementary Information:**

The online version contains supplementary material available at 10.1186/s13619-025-00264-8.

## Background

Among skin flaps for covering soft tissue defects, the random-pattern skin flap is one of the most frequently used in clinics for its simplicity, convenience reliability (Hashimoto et al. 2016). In a random skin flap, oxygen and nutrients were supplied from the microvascular network at the proximal junction with the connecting tissue (Zhou et al. 2019). However, when the length-to-width ratio of the flap is greater than 2:1, the distal portion is prone to be necrotic due to insufficient blood supply (Li et al. 2021). This ischemic necrosis severely affects the therapeutic efficacy of skin flaps in clinical practice. Therefore, finding an effective approach to promote angiogenesis in the flap is important in healing soft tissue defects.

Angiogenesis is critically regulated by hypoxia-inducible factor (HIF)- 1α, which functions as a key transcriptional activator of multiple pro-angiogenic genes, including vascular endothelial growth factor (VEGF)-A (Ramakrishnan et al. 2014). On the other hand, activation of HIF-1α and VEGF is strictly controlled by the PI3K/Akt pathway during angiogenesis. Thus, through the PI3K/Akt/HIF-1α/VEGF signaling axis survival rate of random skin flaps was enhanced by local administration of tetrahydropalmatine (Yang et al. 2018). Demonstrated that tetrandrine enhances the viability of random skin flaps by promoting angiogenesis in flap via the PI3K/Akt signaling pathway. Therefore, augmenting PI3K/Akt pathway might be an effective method to improve skin flap survival via HIF-1α and VEGF-mediated angiogenesis.

Glutamine, the most abundant free amino acid in the circulation and in intracellular pools, serves as a substrate to satisfy energy metabolism in proliferating cells. Intracellular glutamine is converted to glutamate, which is then further converted to alpha-ketoglutarate (α-KG), thus entering the tricarboxylic acid (TCA) cycle (Bodineau et al. 2022). Existing studies have shown that α-KG is significant in protein synthesis, lipid biosynthesis, conditions of immune system homeostasis, and cell death (Wu et al. 2016). Guy Eelen et al. reported that glutamine metabolism is critical for EC motility and migration, which contributes to the formation of new blood vessels in damage (Eelen et al. 2018). Peifeng Hou et al. found that dimethyl-2-ketoglutarate (DKG), the precursor of a key glutamine metabolic, a-ketoglutarate (α-KG), could activate HIF-1α (Hou et al. 2014). These findings led us to speculate whether angiogenesis of skin flaps is associated with ketoglutarate in glutamine metabolism. Neither the changes nor the functions in glutamine metabolism and α-KG have been elucidated in random-pattern skin flaps. Therefore, we focused our attention on the role of α-KG in regulating blood supply and promoting angiogenesis in skin flaps.

In this study, we found that abnormal glutamine metabolism and reduced α-KG occur in random-pattern skin flaps. We used an in vivo assay to explore whether α-KG promotes flap viability in mice and identify the pharmacological targets in vitro. Our results demonstrate that α-KG significantly improves the viability of random-pattern skin flaps by promoting angiogenesis via the PI3K/Akt/HIF-1α signaling pathway, providing new insights into the metabolic regulation of flap survival.

## Results

### RNA-sequencing analysis of skin flaps

**Fig. 1:**
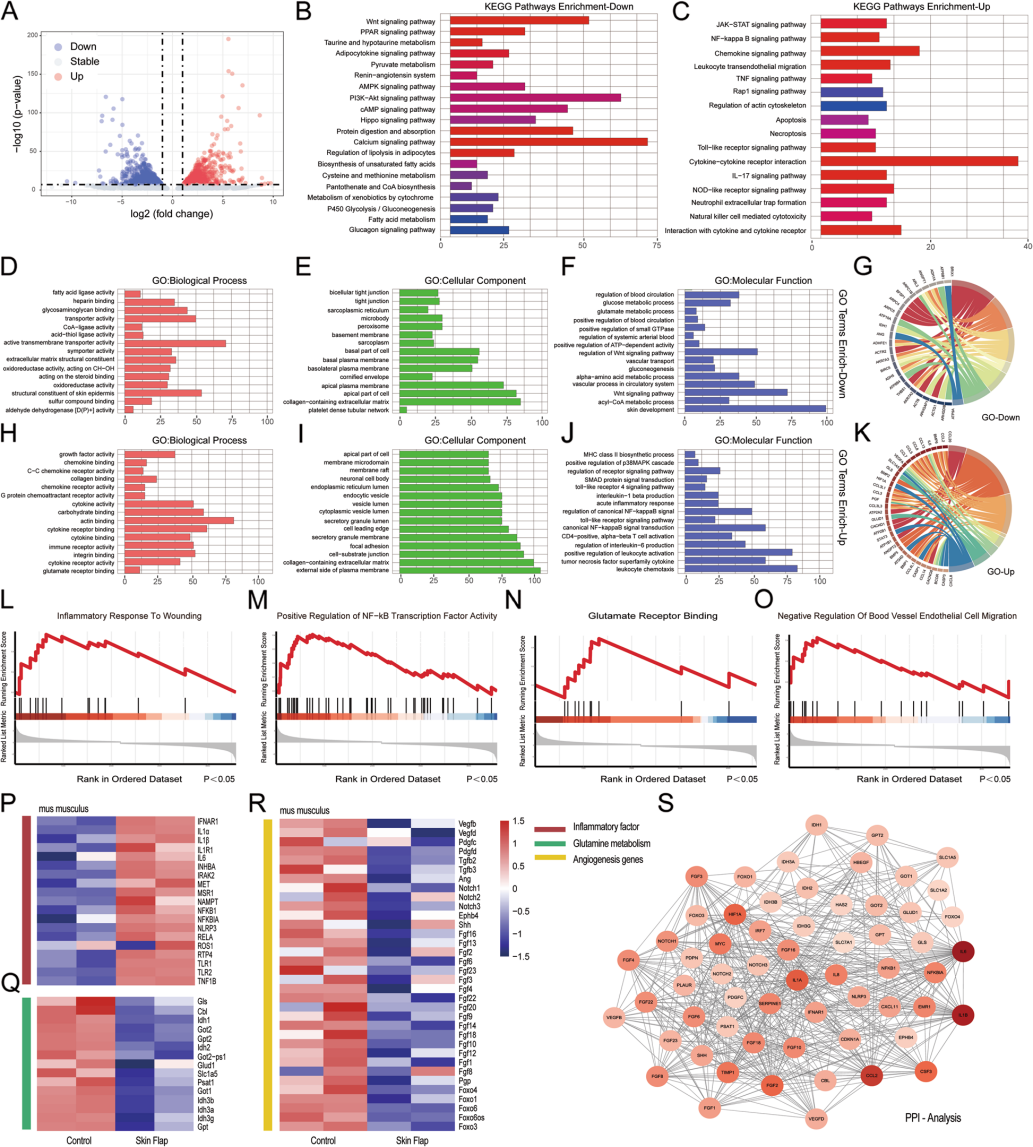
RNA-sequencing analysis of differentially expressed genes (DEGs) between distal portion of random skin flap and normal skin. (**A**) Volcano plot of the transcriptional landscape of differential gene expression. (**B**, **C**) KEGG enrichment analysis of DEGs. The horizontal axis length corresponds to the number of enriched genes. (**D**-**K**) Enrichment analysis of Gene Ontology (GO) terms for DEGs. (**L**-**O**) GSEA comparison of the enrichment of inflammatory, glutamine metabolism and angiogenesis. (**P**-**R**) Heat map illustrating genes related to inflammatory, glutamine metabolism and angiogenesis. Red indicates relatively high expression, while blue signifies low expression. (**S**) PPI network analysis of the interaction of inflammation, glutamine metabolism and angiogenesis-related proteins

Survival of skin flaps is a multifaceted process involving ischemia, inflammation, tissue repair, and angiogenesis events (Jia et al. 2024). To elucidate the complex biological mechanisms underlying survival and pathological necrosis of random skin flap, we performed RNA-sequencing to profile differentially expressed genes (DEGs) at the whole-transcript level. Samples were collected from normal skin and the distal portion of the random skin flap, in which area progressive ischemia occurs at 3 days post-surgery. As shown in the volcano plot (Fig. 1A), in total 5,786 DEGs were identified, comprising 2,992 up-regulated and 2,786 down-regulated genes, respectively. KEGG pathway analysis indicated pronounced differences between normal skin and skin flap tissue. Specifically, inflammatory and immune response pathways were up-regulated, whereas metabolic and cellular signaling pathways were down-regulated in skin flap (Fig. 1B, C). Furthermore, Gene Ontology (GO) enrichment analysis highlighted a marked reduction in one-carbon metabolism, such as glucose and glutamate metabolism, suggesting active metabolic remodeling during flap survival (Fig. 1D-F, H-J). Key terms and pathways from GO and KEGG analyses were further visualized using Circos plots (Fig. 1G, K). Moreover, gene set enrichment analysis (GSEA) displayed significant enrichment in pathways such as inflammatory response to wounding, positive regulation of NF-κB transcription factor activity, glutamate receptor binding, and negative regulation of blood vessel endothelial cell migration in skin flap (Fig. 1L-O). Heatmap analysis confirmed up-regulation of inflammation-related genes, such as *IL1α/β, IL6,* and *NFκB1* (Fig. 1P). Conversely, genes involved in glutamine metabolism including *Slc1a5*(solute carrier family 1 member 5), *Gls* (glutaminase) and *Glud1*(glutamate dehydrogenase 1), as well as angiogenesis including *VEGF-B/C* (vascular endothelial growth factor B/C), *PDGF-C/D* (platelet-derived growth factor C/D) and *Notch 1/2/3* (notch receptor 1/2/3), were down-regulated (Fig. 1Q, R). Protein–Protein Interaction (PPI) network analysis demonstrated strong correlation among inflammation, glutamine metabolism and angiogenesis (Fig. 1S). These findings indicate that under inflammatory conditions, skin flaps exhibit transcriptome-wide down-regulation of glutamine metabolism and angiogenesis.

### Glutamine metabolism was impaired in necrotic random skin flap

**Fig. 2:**
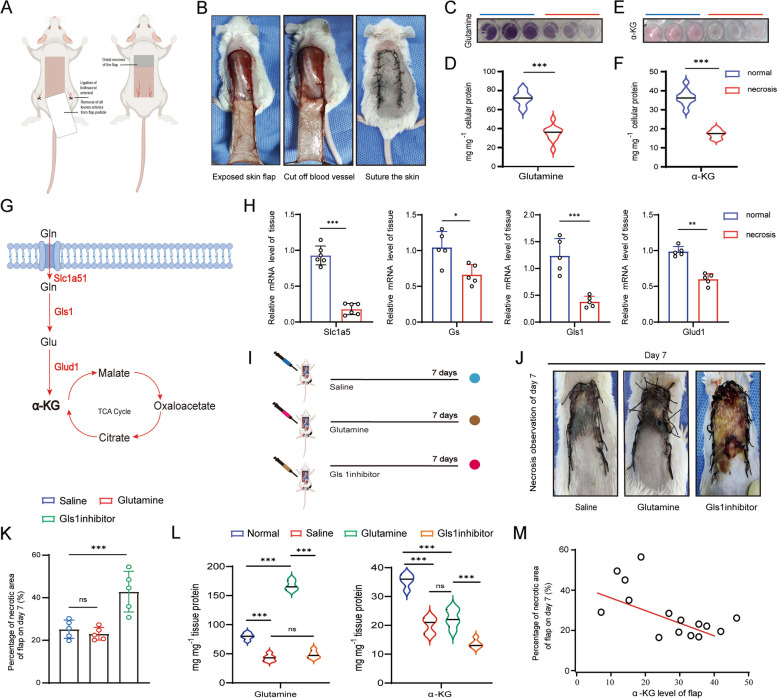
Remodeling of glutamine metabolism in necrotic random skin flaps. (**A**) Schematic diagram of skin flap model. (**B**) Surgical creation of ischemic random skin flap on dorsum of mice. (**C**-**F**) Measurement of glutamine (**C**, **D**) and α-KG (**E**, **F**) concentration in normal and necrotic skin flap. Detection using kits. (**G**) Schematic illustration of pathways related to α-KG metabolism. (**H**) The relative mRNA level of Slc1a5, Gs, Gls1 and Glud1 in normal and necrosis skin flap 7 Days after surgery; *n* = 5. (**I**) Saline, Glutamine and Gls 1 inhibitor treated mice for 7 days. (**J**) Gross observation of skin flap injected with saline, glutamine group and Gls 1 inhibitor BPTES on day7 after surgery. (**K**) Percentage of necrotic area in saline-, glutamine-, and and Gls 1 inhibitor- treated skin flap on day 7. (**L**) Measurement of glutamine and α-KG level in normal, saline-, glutamine-, and Gls 1 inhibitor-treated skin flaps. (**M**) Correlation analysis of percentage of necrotic area with α-KG levels in skin flap on day 7. *p < 0.1, **p < 0.01, ***p < 0.001, ****p < 0.0001

Following skin flap elevation, survival of the skin flap was found to depend primarily on the blood supply from flap pedicle (Fig. 2A). A random flap model was established by ligating bilateral vessels and excising all identifiable arteries from the pedicle, followed by in situ suturing (Fig. 2B). Quantification measurements showed that levels of glutamine and α-KG in necrotic part decreased nearly 2 folds compared to those in the normal tissue (Fig. 2C-F). In the glutamine metabolism pathway, extracellular glutamine is transported into cells by Slc1a5*,* and then subsequently converted to glutamate by Gls1, and to α-KG by glutamate dehydrogenase 1 (Glud1), ultimately enter the TCA cycle (Hensley et al. 2013) (Fig. 2G). We found that mRNA levels of major molecules that regulate glutamine metabolism including *Slc1a5*, *Gs* (glutathione synthetase), *Gls1,* and *Glud1* were significantly reduced in necrosis flap (Fig. 2H). To examine whether disrupted glutamine metabolism contributes to skin flap necrosis, we administered BPTES, an inhibitor of GLS1, daily for 7 days starting immediately after surgery in the flap (Fig. 2I). BPTES treatment resulted in an approximately two-fold increase in the necrotic area compared with the normal and saline control group. In contrast, administration of glutamine displayed no detectable improvement in viability of skin flap (Fig. 2J). Further analysis showed that BPTES administration did not significantly alter glutamine levels but led to a pronounced reduction in α-KG (Fig. 2K). Moreover, the reduced α-KG level was positively correlated with an increase in skin flap necrosis (Fig. 2L, M). Together, these results indicated that impaired glutamine metabolism leads to reduced α-KG levels, which contributes to the progression of necrosis in random skin flaps.

### α-KG enhances skin flap survival and improves blood perfusion

**Fig. 3:**
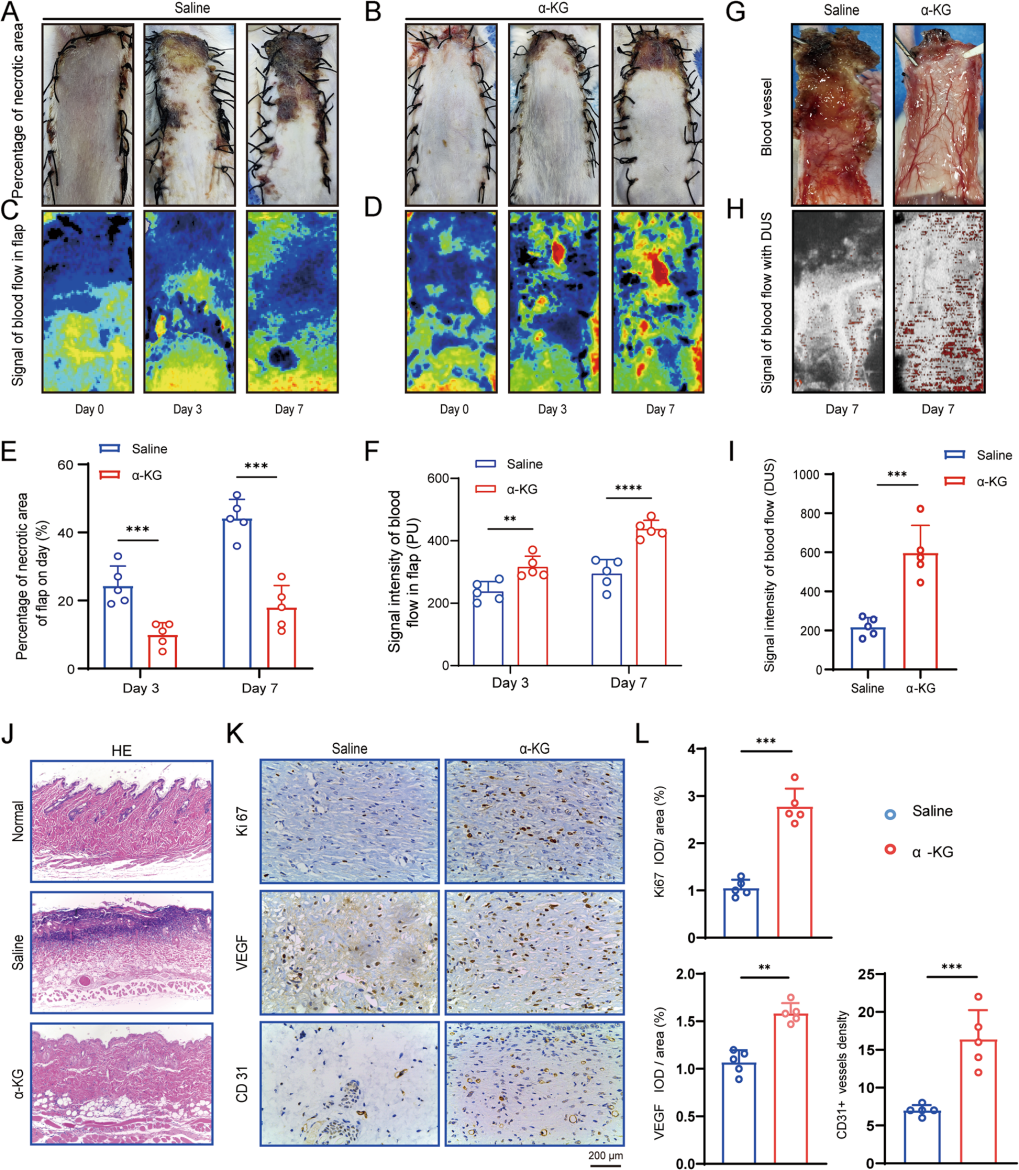
α-KG injection increased survival of skin flap. (**A**-**D**) Gross observation (**A**, **B**) and laser Doppler images (**C**, **D**) of skin flap that received saline and α-KG injection, on day 0, 3 and 7 post-surgery. (**E**, **F**) Necrotic area and blood flow in skin flaps was semi-quantified. (**G**-**I**) Vascular network (**G**), signal of blood flow detected with DUS (**H**) and its quantification (**I**) on day 7 post-surgery in saline and α-KG-treated flaps. (**J**) H&E staining of zone Ⅱ of normal, saline- and α-KG-treated skin flap. (**K**) qRT-PCR of Ki-67, VEGF and CD31 in saline- and α-KG-treated flap. (**L**) Immunostaining of Ki-67, VEGF and CD31 in saline and α-KG-treated skin flaps. *p < 0.1, **p < 0.01, ***p < 0.001, ****p < 0.0001

Since local administration of glutamine showed no significant effect on salvaging the skin flap, we instead administered DM-α-KG, a cell-permeable analog of α-KG and an end product of glutamine metabolism, at 0, 3, and 6 days after surgery. Macroscopic examination revealed progressive necrosis in saline-treated flaps, with nearly half of the flap area necrotic by day 7 (Fig. 3A). In contrast, α-KG treatment markedly reduced the necrotic area, limiting it primarily to the distal tip of the flap (Fig. 3B). Quantification confirmed that α-KG administration significantly reduced the necrotic area at 3 days (α-KG: 9.85% vs. saline: 22.72%, *P* < 0.001) and 7 days (α-KG: 17.62% vs. saline: 43.59%, *P* < 0.001) post-surgery (Fig. 3E). Meanwhile, Laser Doppler imaging demonstrated that α-KG treatment markedly improved blood flow within the flap, achieving approximately three times the perfusion level of the saline-treated group (Fig. 3C, D, F). By 7 days, well-organized vascular networks were evident in α-KG-treated flaps, whereas saline-treated flaps exhibited signs of hemorrhage and thrombosis (Fig. 3G). Consistent with these observations, Ultrasound Microvascular Imaging revealed that the blood flow signal intensity (DUS) in α-KG-treated flaps was three times higher than in saline-treated flaps (Fig. 3H, I). Histological observation further supported these findings, showing extensive necrosisin saline-treated flap, which was substantially ameliorated by α-KG treatment (Fig. 3J). In line with the enhanced perfusion, α-KG administration significantly upregulated the expression of angiogenesis-related markers, including Ki-67, VEGF, and CD31 which was further confirmed by immunohistochemical staining (Fig. 3K, L). Collectively, these results demonstrated that local administration of α-KG improved survival of ischemic flap by, at least partially through increasing blood perfusion.

### α-KG enhanced expression of VEGF and HIF-1α in endothelial cells

**Fig. 4:**
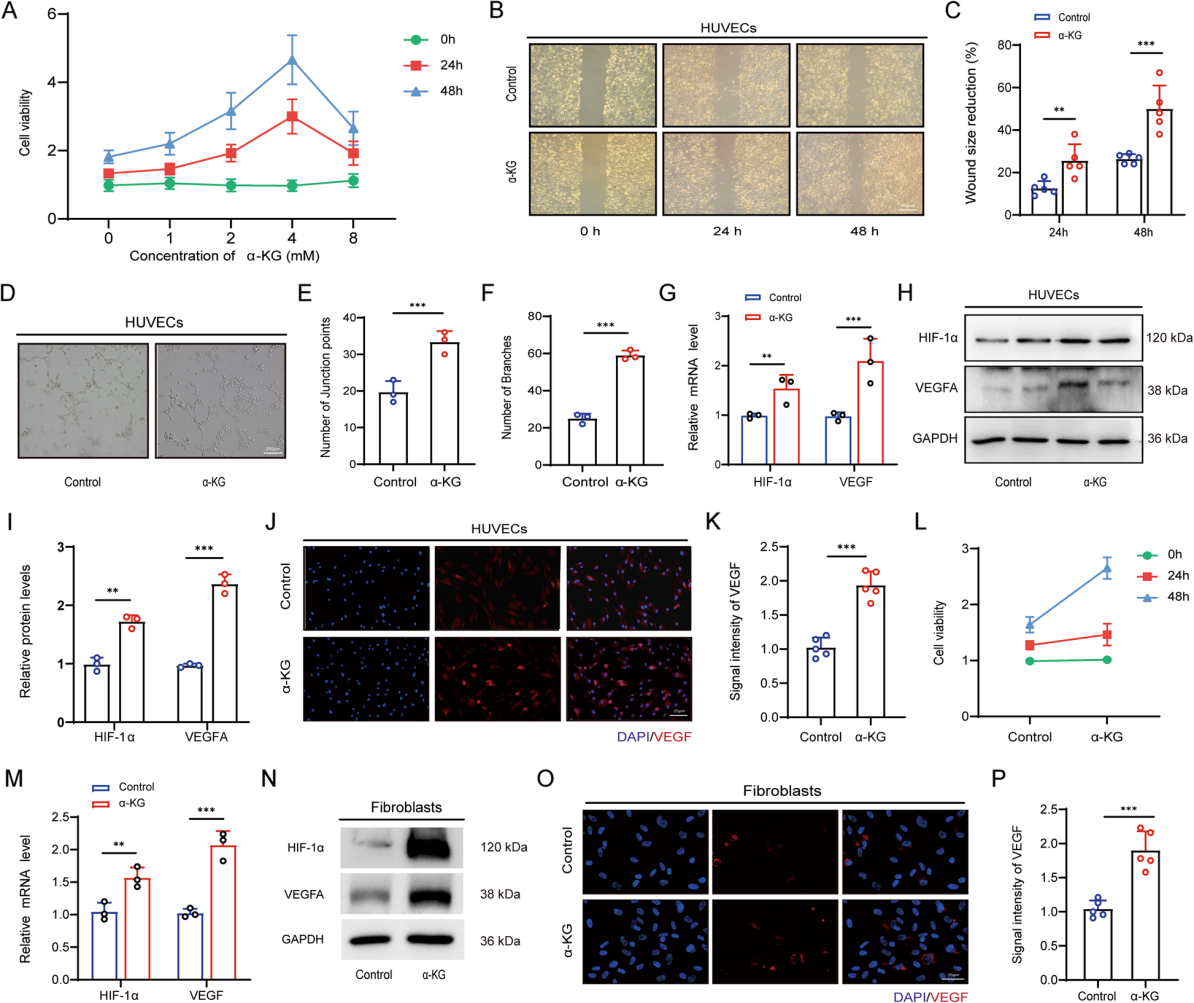
Proangiogenic effect of α-KG on human umbilical vein endothelial cells (HUVECs) and dermal fibroblasts. (**A**) Cell viability of HUVECs at different α-KG concentrations. (**B**) Cellular migration assay of HUVECs treated with or without α-KG at 24, 48 h. (**C**) Quantitative analysis of wound size reduction. (**D**) In vitro tube-formation assay of α-KG-treated and non-treated HUVECs for 48 h. (**E**, **F**) Quantification of junction points (**E**) and branches (**F**). (**G**, **H**, **I**) RT-PCR (**G**), western blot (**H**) and quantitative analysis of relative protein levels (**I**) of HIF-1α and VEGF in α-KG-treated and non-treated HUVECs; *n* = 5. (**J**, **K**) Immunofluorescent staining (**J**) and relative intensity (**K**) of VEGF in HUVECs treated with or without α-KG for 48 h. (**L**) Cell viability of dermal fibroblasts treated with α-KG. (**M**, **N**) RT-PCR (**M**) and western blot (**N**) of VEGF and HIF-1α in fibroblasts. *n* = 5. (**O**, **P**) Immunofluorescent staining (**O**) and relative intensity (**P**) of VEGF in fibroblasts in the presence or absence of α-KG for 48 h

Given that angiogenesis was markedly increased in α-KG treated skin flap, we further investigated the effect of α-KG on the functional activities of HUVECs. Treatment with varying concentrations of α-KG (0, 1, 2, 4, 8 mM) for 48 h, we found that HUVECs had the highest viability at a concentration of 4 mM. Thus, we chose α- KG at the doses of 4 µM in our subsequent experiments (Fig. 4A). Wound healing assays demonstrated that α-KG treatment significantly enhanced the migratory capacity of HUVECs compared to untreated controls (Fig. 4B, C). Moreover, α-KG exposure led to an approximately 30% increase in tubular structure formation when HUVECs were cultured in three-dimensional gels (Fig. 4D-F). VEGF expressed in endothelial cells plays a critical role in regulating angiogenesis, which was tightly modulated by HIF-1α at the transcriptional level under ischemic conditions (Chang et al. 2007). We thus treated HUVECs with α-KG for 48 h and our findings showed that the level of VEGF and HIF-1α was markedly elevated, at both mRNA (Fig. 4G) and protein level (Fig. 4H, I). Immunofluorescence staining further confirmed upregulation of VEGF expression in α-KG-treated HUVECs (Fig. 4J, K). Based on previous findings that dermal fibroblasts promote angiogenesis in ischemic flaps via paracrine secretion of VEGF (Li et al. 2024b). We also examined whether α-KG exerts a pro-angiogenesis response in dermal fibroblasts. We found that α-KG significantly increased the expression of *VEGF* and *HIF-1α* in fibroblasts at a concentration of 4 mM for 48 h, which exhibited the highest cellular viability (Fig. 4L), as determined by RT-PCR and Western blotting (Fig. 4M, N). Accordingly, increased expression of VEGF was further observed in α-KG-treated fibroblasts by immunofluorescent staining (Fig. 4O, P).

### α-KG activated PI3K/Akt/HIF-1a axis in endothelial cells

**Fig. 5:**
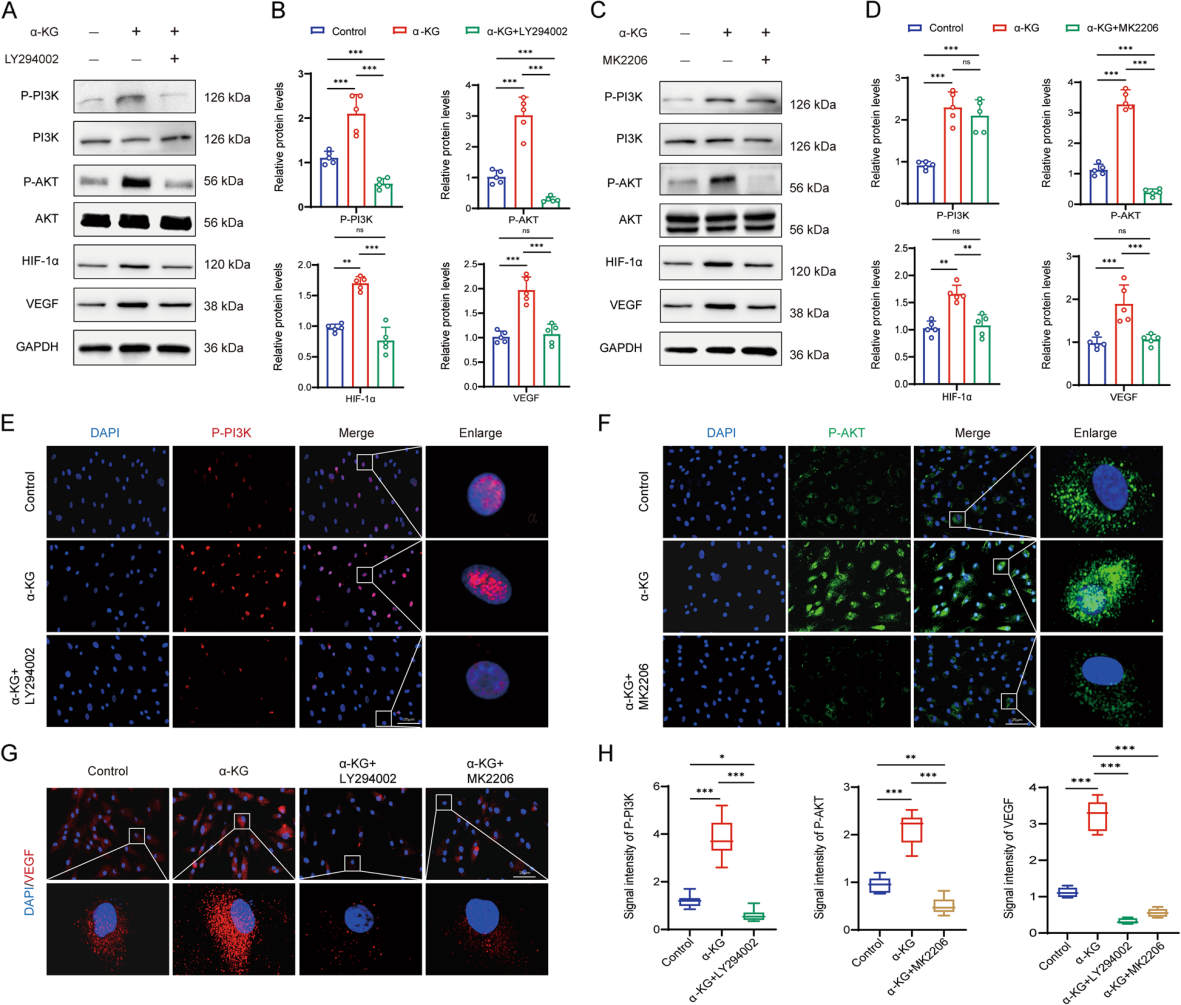
α-KG triggered activation of PI3K-AKT pathway in HUVECs. (**A**, **B**) Western blot and its quantification of P-PI3K, PI3K, P-AKT, AKT, HIF-1α, VEGF in HUVECs treated with or without α-KG in the presence or absence of LY294002. Values were normalized to GADPH levels; *n* = 5. (**C**, **D**) Western blot and its quantification of P-PI3K, PI3K, P-AKT, AKT, HIF-1α, VEGF in HUVECs treated with or without α-KG in the presence or absence of MK-2206. Values were normalized to GADPH levels; *n* = 5. (**E**, **F**) Immunofluorescentstaining for P-PI3K (E, P-PI3K, red) and P-AKT (F, P-AKT, green) in HUVECs treated with or without α- KG in the presence or absence of LY294002 or MK-2206, respectively. (**G**) Immunostaining for VEGF (VEGF, red) in normal, α-KG-treated HUVECs in the presence or absence of LY294002 or MK-2206. *n* = 5. (**H**) Relative fluorescence intensity of P-PI3K, P-AKT and VEGF. *p < 0.1, **p < 0.01, ***p < 0.001, ****p < 0.0001

To further evaluate whether the proangiogenic effect of α-KG relies on activation of PI3K/Akt signaling, which has been linked with upregulation of HIF-1α, we treated HUVECs with α-KG for 48 h. We found that α-KG stimulation resulted in a significant increase in phosphorylation of PI3K and Akt, along with upregulated expression of HIF-1α and VEGF in HUVECs (Fig. 5A, B). To further validate the dependence of HIF-1α and VEGF upregulation on PI3K/Akt activation, we inhibited the pathway using specific inhibitors, MK-2206 (Akt inhibitor) and LY294002 (PI3K inhibitor). Both inhibitors markedly suppressed the expression of HIF-1α and VEGF in α-KG-treated HUVECs (Fig. 5A-D). Consistent with these findings, immunofluorescent staining revealed a significant increase in the proportion of cells positive for p-PI3K and p-Akt following α-KG treatment (Fig. 5E, F). Conversely, inhibition with LY294002 or MK-2206 attenuated PI3K/Akt activation and subsequently reduced VEGF expression (Fig. 5G, H). Together, these data demonstrated that α-KG promotes the HIF-1α/VEGF axis through activation of PI3K/Akt signaling in vascular endothelial cells.

### Blockage of PI3K-Akt signaling reduced survival and angiogenesis in skin flap treated with α-KG

**Fig. 6:**
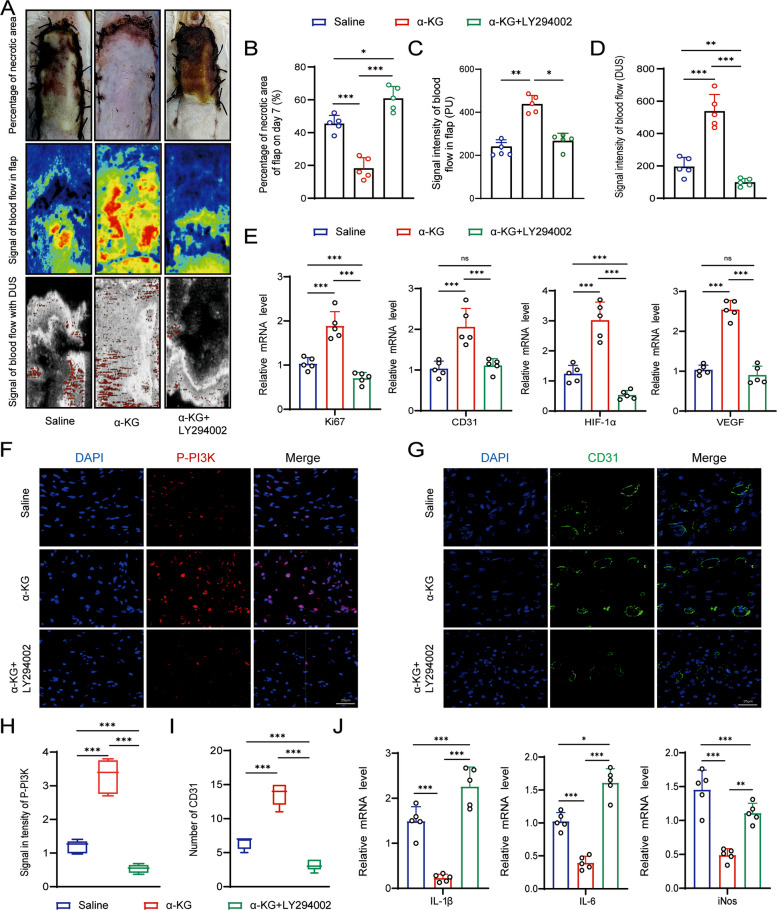
α-KG enhanced survival and angiogenesis via PI3K-AKT signaling in ischemic flap. (**A**) Gross view, laser Doppler and ultrasound microvascular analysis images of saline, α-KG and α-KG + LY294002-treated skin flaps on day 7 after surgery; *n* = 5. (**B**, **C**, **D**) The percentage of necrotic area (**B**), blood flow signal (**C**), and signal intensity of blood flow (DUS) for each flap on day 7. (**E**) RT-PCR of Ki-67, CD31, HIF-1α, and VEGF expression in saline, α-KG and α-KG + LY294002-treated skin flaps; *n* = 5. (**F**-**I**) Immunostaining (**F**) and relative fluorescence intensity (**H**) for P-PI3K (P-PI3K, red) in skin flaps treated with α-KG and α-KG + LY294002. Immunostaining (**G**) and quantification of relative number (**I**) of positive CD31 cells (CD31, green). (**J**) RT-PCR of IL-1β, IL-6, and iNOS expression in saline, α-KG and α-KG + LY294002-treated skin flaps; *n* = 5. *p < 0.1, **p < 0.01, ***p < 0.001, ****p < 0.0001

**Fig. 7:**
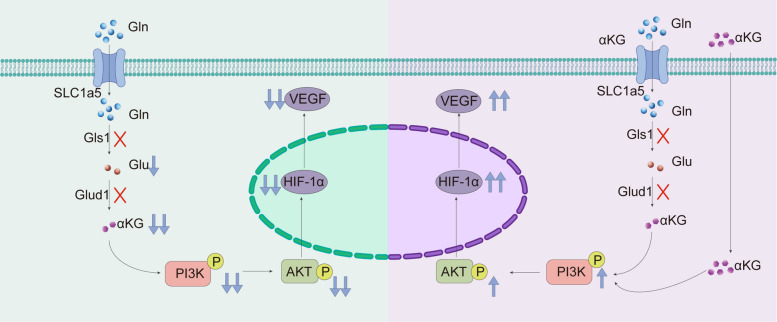
Illustration of the impact of α-KG treatment on skin flaps. In the left panel, the abnormal glutamine metabolism was observed in ischemic flaps characterizing by the downregulation of Gls1, Glu, Glud1, and α-KG. In the right panel, α-KG treatment mitigated ischemic necrosis in flaps by phosphorylating PI3K and AKT, which subsequently lead to the upregulation of HIF-1α and VEGF

To address whether α-KG promoted angiogenesis via activation of PI3K-Akt signaling in random skin flap, we co-administered α-KG and the PI3K inhibitor LY294002 at 0, 3, and 6 days after surgery. With administration of LY294002, survival of the α-KG treated flap was markedly reduced (Fig. 6A). The percentage of necrotic area increased from 17.85% in α-KG treated flap to 63.47% in flap received LY294002 injection, which was significantly higher than that in normal skin flap (42.78%) on the 7th day (Fig. 6B). Consistent with these observations, blood perfusion in the flaps, assessed via laser Doppler imaging and ultrasound microvascular imaging, was significantly compromised by LY294002 administration. Compared to flaps treated with α-KG alone, co-treatment with LY294002 resulted in a 2.52-fold reduction in blood flow signals as detected by laser Doppler (Fig. 6A, C). Similarly, ultrasound microvascular imaging analysis revealed a pronounced decrease in blood flow in α-KG treated flaps with LY294002 (63.48% with LY294002 vs. 17.96% with α-KG and 16.73% in ischemic flaps, *P* < 0.001) (Fig. 6A, D). At the molecular level, immunohistochemical analyses showed that LY294002 administration significantly downregulated the expression of Ki-67, CD31, HIF-1α and VEGF in areas adjacent to the necrotic zone. Conversely, inflammatory markers including IL-1β, IL-6*,* and iNOS were upregulated (Fig. 6E, J). Immunofluorescence staining further confirmed that α-KG strongly activated PI3K signaling in the flaps, an effect that was substantially abolished by LY294002 co-treatment (Fig. 6F, H). Additionally, a significant decrease in CD31-positive cells was observed in the LY294002-treated group (Fig. 6G, I). Collectively, these results demonstrate that the pro-angiogenic and pro-survival effects of α-KG in ischemic skin flaps are mediated primarily through activation of the PI3K–Akt signaling pathway in vivo (Fig. 7).

## Discussion

Necrosis that results from defective angiogenesis and consequently reduced blood perfusion limits the clinical outcomes of skin flap (Fan et al. 2021). Metabolic changes in glycolysis, and fatty acid oxidation, have been documented to play distinct and essential roles in angiogenesis during development and postnatal tissue repair (Rohlenova et al. 2018), respectively. In the present study, we identified the impairment in glutamine metabolism in the necrotic area of random skin flaps, which was associated with reduced capillary density and blood perfusion. With local administration of α-KG, the viability of the skin flap was greatly increased which was in concordance with enhancement in angiogenesis.

Glutamine is the most abundant nonessential amino acid in peripheral circulation and is highly consumed by proliferating ECs to replenish the TCA cycle as an anaplerotic source of carbons for protein and nucleotide synthesis during vessel sprouting (Stephan et al. 2023; Wu et al. 2019). Deficiency in glutamine metabolism, as reported by Huang et al. (Huang et al. 2017), resulted in vessel sprouting defects due to impaired proliferation and migration of ECs. However, whether impairment of glutaminolysis is involved in angiogenesis defects under pathological milieu remains largely unknown. In the present study, by bioinformatic analysis of RNA sequencing data, we identified significant abnormalities in glutamine metabolism within the necrotic skin flaps, highlighting its critical role in angiogenesis. By the analysis, the expression of key molecules regulating glutamine metabolism, such as *Slc1a5, Gls1,* and *Glud1*, was significantly reduced, along with decreased glutamine levels and capillary density in necrotic skin flap. More importantly, topical administration of Gls1 inhibitor led to a remarkable increase in necrotic area as a result of reduced angiogenesis in the skin flap. Thus, deficiency in glutamine metabolism contributes largely to necrosis of the skin flap that is induced by a decrease in angiogenesis. It is interesting to find out that local glutamine repletion failed to restore normal glutamine metabolism in the skin flap. As above mentioned, expression of both Slc1a5 and Gls1 was reduced in a necrotic flap, which might resulted in a decrease of intracellular transportation of glutamine and a reduction in replenishing of glutamine into the tricarboxylic acid cycle, respectively, indicating that supplementation of downstream metabolite of glutamine metabolism rather than extracellular glutamine itself would rescue defects in glutamine pathway in necrosis tissue.

By administration of DM-α-KG, we found that the proliferation, migration, and tube-formation capacity of HUVECs was largely improved, an effect which has been attributed to enhancements in TCA cycle anaplerosis, macromolecule production, and redox homeostasis (Kim et al. 2017). In vivo, DM-α-KG treatment resulted in the restoration of blood flow and an increase of microvascular density in the skin flap, showing a pro-angiogenic outcome in ischemic flap tissue. It is noteworthy that, in the presence of DM-α-KG, stability HIF-1α was promoted in both HUVECs and fibroblasts. During the repair of damaged tissue, HIF-1α is a critical player in promoting the transcription of downstream target genes that regulate angiogenesis and vascular remodeling. Under normoxia, a stable level of α-KG, together with ascorbate and Fe(II), has been linked with initiation of HIF-1α degradation by acting as cofactors of prolyl hydroxylase domain (PHD)-containing proteins. For instance, Joyal et al. (Joyal et al. 2016) demonstrated that lowing α-KG levels promotes the stabilization of HIF1α which subsequently leads to neovascularization in mice retinas. However, it seems that cell-permeable DM-α-KG elicited a different effect in stabilizing HIF-1α compared with α-KG. Unlike a-KG and Octyl-2KG (an analog of a-KG), it has been reported that DM-α-KG stabilized HIF-1a by inhibiting PHD in human dermal fibroblasts and mammary epithelial MCF-10A cell line under normoxia, an effect has been supposed to be attributed to increased production of succinate and fumarate, which are the downstream metabolites of a-KG in TCA cycle that inhibits the activity PHD2 (Hou et al. 2014). However, whether the effect of DM-α-KG on activating HIF-1α depends on divergent pathways other than the accumulation of succinate and fumarate in ECs remains to be explored.

Given that activation of the PI3K/Akt pathway has been linked with the enhancement of VEGF production through either HIF-1α-dependent or independent mechanisms (Zhong et al. 2000), we addressed whether the addition of DM-α-KG increased the activation of the PI3K/Akt signaling pathway in HUVECs. The role of α-KG in modulating PI3K/Akt signaling is controversial. In human glioblastoma cells, α-KG has been shown to enhance PDPK1 transcription by reducing the inhibitory histone modification H3K27me3, thereby promoting the activation of the PI3K/Akt/mTOR pathway (Yang et al. 2025). Conversely, in bone marrow-derived mesenchymal stem cells, a decrease in α-KG levels results in increased trimethylation of histones H3K9 and H3K27, which activates the PI3K/Akt pathway (Wang et al. 2022). Our findings demonstrated significant activation of PI3K/Akt in HUVECs treated with DM-α-KG, along with increased stabilization of HIF-1α and elevated *VEGF* expression. Accordingly, the stabilization of HIF-1α induced by DM-α-KG was significantly diminished when either PI3K or Akt signaling was inhibited, both in vitro and in vivo. Under normoxia, HIF-1α is typically hydroxylated by PHD enzymes, recognized by the von Hippel-Lindau protein (pVHL) for ubiquitination and subsequent degradation. However, it has been reported that the activation of the PI3K/Akt pathway resulted in the inhibition of PHD activity, thereby reducing the hydroxylation and degradation of HIF-1α (García-Maceira and Mateo 2009; Maxwell et al. 1999). Furthermore, activation of mTOR, a downstream effector of PI3K/Akt, has contributed to the increase in the translation and stability of HIF-1α.

In conclusion, our study identified a disruption in glutamine metabolism in ischemic random pattern skin flap, which is characterized by reduced levels of Gln and α-KG. We revealed that local administration of DM-α-KG largely improved the survival area of the ischemic flap with increased blood flow and microvascular density. The pro-angiogenesis effect of DM-α-KG can be attributed to the promotion of HIF-1α stability which was mediated by the activation of the PI3K/Akt pathway in HUVECs.

## Materials and methods

### Animals

A total of thirty 8-week-old SPF-grade BALB/C mice(weighing 20 ± 2 g) were purchased from the Shanghai SLAC Laboratory Animal Co. Animals were kept in individual cages under standardized SPF-grade conditions in the animal center of Tongji University School of Medicine. All animal experimental procedures were approved by the Animal Care and Experimentation Committee of the School of Medicine, Tongji University, Shanghai, China.

### Establishment of the ischemic random pattern skin flap

To establish the ischemic skin flap, mice were anesthetized via intraperitoneal injection of 1% sodium pentobarbital at a dosage of 50 mg/kg. The modified McFarland flap technique was employed to create random pattern flaps on the dorsum of mice (Lee et al. 2017). Briefly, hair on the dorsum of mice was removed using an electric razor and depilatory cream. Subsequently, a random skin flap that had its pedicle on the caudal dorsum, in a size of 1.5 × 4.5 cm^2^, was elevated with intact subcutaneous deep fascia. Following the transaction of all known arteries at the pedicle of the flap, flaps were then promptly sutured back onto the wound bed using 4–0 non-absorbable sutures. The entire flap was divided from caudal to cephalic into three equal zones: zone I (proximal zone), zone II (intermediate zone), and zone III (distal zone).

In each animal study, six mice were randomly included and subjected to subcutaneous injection at a dosage of 1 mL on days 0, 3, and 6 after surgery, respectively. In addition, a control group in which a normal flap was created with intact blood supply, was included. Animals in experimental group received injection of Gln (L- Glutamine, 1g/kg, Sigma, USA), α-KG (DM-α-KG, 500 mg/kg, Sigma, USA), BPTES (BPTES, 12.5 mg/kg, Sigma, USA) and α-KG + LY294002 (a combination of DM-α-KG at 500 mg/kg and LY294002 at 50 mg/kg, Sigma, USA), respectively (Li et al. 2024a; Li et al. 2025). Mice injected with saline served as control. After 7 days of treatment, all mice were sacrificed and skin flaps were harvested for further analysis.

### Gross observation and assessment of flap survival

The appearance, color, and shrinkage that characterizes necrosis of the flap were assessed on days 0, 3, and 7 post-operation. The percentage of flap survival area was assessed on day 7 using Image J software (version 6.2; Media Cybernetics). Survival area (%) was calculated as follows: survival percentage = surviving area/total area × 100%.

### Laser Doppler Blood Flow (LDBF) and Ultrasound Microvascular Imaging

On day 7 post-operation, mice were anesthetized and the entire flap was scanned using the LDBF imaging system (Moor Instruments Ltd, moorVMS-LDF, United Kingdom). The data collected from scans were quantified using moorLDI (version 6.1) and the intensity of blood flow was represented as perfusion units (PUs). Individual animals underwent three equal scans at each time point.

On day 7 post-surgery, mice were subjected to scanning with a high-frequency transducer in the VisualSonics VEVO LAZR-X ultrasound system (Fujifilm VisualSonics Inc., Ontario, Canada). Blood flow was quantified by Vevo Lab (VisualSonics) and the mean value for each flap was calculated by equal three scans of individual animals.

### Histological analysis

On day 7, tissue specimens (1 cm × 1 cm) were harvested from zone II of each flap for histological examination. Specimens were fixed in 4% paraformaldehyde for 24 h, embedded, sectioned, and stained with hematoxylin and eosin (H&E) staining. Histological changes were observed under a light microscope (Leica, Wetzlar, Germany).

Immunohistochemistry was performed using the following primary antibodies against Ki-67, CD31, and VEGF (all from Abcam, 1:1000), respectively. HRP-labeled secondary antibodies (goat anti-rabbit or goat anti-mouse, ZSGB-BIO, China) were added and the integrated absorbance (OD) values of Ki-67, CD31, and VEGF were calculated using Image-Pro Plus software. The numbers of microvessels per unit area (/mm^2^) in each section were calculated. Immunofluorescent staining was performed using the following primary antibodies against P-PI3K and CD31 (both from Abcam, 1:1000), followed by FITC conjugated secondary antibodies (goat anti-rabbit or goat anti-mouse, ZSGB-BIO, China), and counterstained with DAPI, examined under a fluorescence microscope (Leica SP8, Germany).

### Glutamine and alpha-ketoglutarate content assay

To assess the levels of glutamine (Gln) and alpha-ketoglutarate (α-KG) in a flap, a sample weighing 20 mg was homogenized with a micro homogenizer in 100 µL of cold lysate and then centrifuged at 13,000g for 10 min. Levels of α-KG and Gln in supernatant were measured using α-KG assay kit (Abcam, ab83431), and glutamine assay kit (Abcam, ab197011), respectively. The concentration of Gln and α-KG was calculated according to parameters at an absorbance of 570 nm.

### Cell culture

Human umbilical vein endothelial cells (HUVECs) and dermal fibroblasts were maintained in Dulbecco's modified Eagle's medium (Gibco, USA) containing 10% fetal bovine serum (HyClone, USA) and 1% penicillin/streptomycin (Gibco, USA). Cells were treated with α-KG (2uM, Sigma, USA), LY294002(inhibitor of PI3K, 10 µM, Sigma, USA), and MK-2206 (inhibitor of Akt, 100 nM, Sigma, USA) for 48 h, respectively.

The proliferation of HUVECs treated with α-KG (2uM) was assessed using a CCK8 assay kit (Dongren, China) at 24, and 48 h after seeding according to the manufacturer’s instruction (Ge et al. 2018; Zhang et al. 2021). Tube formation assay was carried out by culturing HUVECs on glass slides coated with endothelial cell matrix gel solution (BD Biocoat, NJ, USA). Several tubes were examined under a phase contrast microscope (Olympus Inc., Tokyo, Japan), and tubes in randomly selected areas of each well were counted. The migration of cells under different conditions was investigated with a wound healing assay by creating a scratched wound using a 200-µl pipette tip. Changes in the width of the scratched wound were analyzed with Image J software (version 6.2; Media Cybernetics) after 24, and 48 h.

Intracellular expression of VEGF, P- PI3K, and P-Akt was detected by adding primary antibodies against VEGF, P-PI3K, and P- Akt (all from Abcam, 1:1000) at 4 °C, followed by secondary antibodies (goat anti-rabbit or goat anti-mouse, ZSGB-BIO, China) conjugated with FITC. After counterstaining with DAPI (Beyotime, C1005, China), cells were observed under a fluorescent confocal microscope (Leica SP8, Germany).

### RNA sequencing and analysis

Total RNA was extracted from the distal portion of the random skin flap and normal skin using TRIzol reagent (Invitrogen, USA). Sequencing libraries were constructed according to the Illumina NovaSeq 6000 User Guide, where relative gene expression levels were calculated using Cufflinks v2.2.1 in fragments per kilobase per million reads (FPKM). Transcript annotation files were obtained from the Gencode v22 Comprehensive Gene Annotation gtf file (http://www.gencodegenes.org). The Bioconductor software package edgeR was used to identify differentially expressed genes (DEGs) between the distal portion of a random skin flap and normal skin. The differential gene screening criteria in this project were: corrected *P* value < 0.05 and Log2FC ≥ 1.5 and any set of FPKM > 1. Specifically, differential pathway enrichment analysis was carried out directly using the Gene Ontology (GO), Gene Set EnrichmentAnalysis (GSEA), and Kyoto Encyclopedia of Genes and Genomes (KEGG) databases, and visualization of signaling pathways to draw Volcano Plot and heat maps.

### RNA isolation and RT-PCR

For the mRNA expression analysis, skin flap tissues from zone II were collected and then homogenized using a cryogenic grinder (Shanghai Jingxin JXFSTPRP-CL) in RNAiso Plus (9108; Takara). Total RNA was extracted from cells using TRIzol reagent (Invitrogen, USA), and the concentration along with the quality of the extracted RNA was evaluated with a NanoDrop ND-1000 Spectrophotometer (Agilent, USA). Following RNA extraction, complementary DNA (cDNA) was synthesized using the.

PrimeScript RT Master Mix (RR036A, Takara). Real-time PCR was subsequently performed using TB Green Premix Ex Taq II (RR820A; Takara) on a QuantStudio 7 Flex real-time PCR system (Thermo Fisher Scientific). The primer sequences for all genes analyzed in this study are detailed in Table S1. Gene expression levels were quantified using the ΔΔ − Ct method, with β-actin as the housekeeping gene and the control group serving as the reference samples.

### Western blotting

To detect protein content, cells were lysed on ice for 15 min using RIPA buffer supplemented with protease and phosphatase inhibitors (Beyotime, P1045). The protein concentration was then measured using the BCA assay (Beyotime, P0012). Equal amounts of protein were separated by 12.5% (w/v) gel electrophoresis and subsequently transferred onto PVDF membranes (Millipore). Membrane blocking was performed at approximately 25 °C using 5% defatted milk powder (Beyotime, P0216). The membranes were then incubated at 4 °C for 24 h with primary antibodies against HIF-1α, VEGF, GAPDH, PI3K), P-PI3K, Akt, and P-Akt (all from Abcam, 1:1000). Following this, the membranes were washed with TBS buffer containing Tween and incubated with a secondary antibody at a dilution of 1:5000 for 2 h at approximately 25°C. Protein bands on the membranes were visualized using the ECL Plus Reagent Kit. The intensity of the bands was quantified using Image J software (version 6.2; Media Cybernetics).

### Statistical analyses

The statistical analysis of the data was conducted using GraphPad Prism 9.5 software (GraphPad Software Inc., San Diego, CA, USA). Results are presented as mean ± standard deviation (SD). To compare mean values between the two groups, an independent-sample t-test was employed. Significance levels were set at *p < 0.05, **p < 0.01, ***p < 0.001, and ****p < 0.0001.

### Materials

**Table:** 

REAGENT or RESOURCE	SOURCE	IDENTIFIER
Antibodies		
Ki67 Antibody	abmart	TW0001
CD31 antibody	abmart	TB5008
VEGF Antibody	abmart	T55096
VEGFA Antibody	abmart	TA5131
HIF-1-alpha Antibody	abmart	T55824
GAPDH Antibody	abmart	P60037
PI3 Kinase p85 alpha Antibody	abmart	T40115
Phospho-PI3-kinase p85- alpha/gamma (Tyr467/199) pAb	abmart	T40116
AKT Antibody	abmart	PA1036
Phospho-Akt (Ser473) Antibody	abmart	T40067
IL1 beta Antibody	abmart	TA5103
IL6 Antibody	abmart	TD6087
Chemicals		
α-KG	sigma-aldrich	75890
glutamine	sigma-aldrich	1,294,808
gls1 inhibitor	sigma-aldrich	CB-839
LY294002	sigma-aldrich	440,202
MK2206	sigma-aldrich	124,005
α-KG assay kit	abmart	ab83431
glutamine assay kit	abmart	ab197011

## Supplementary Information

Supplementary Material 1. Table S1. Primers used in this study.

## Data Availability

The RNA sequencing data generated and analyzed during the current study are available in the NCBI BioProject repository under the accession number PRJNA1359387. All other data and materials supporting the findings of this study are available from the corresponding author upon reasonable request

## Abbreviations

α-KG: Alpha-ketoglutarate
HIF-1α: Hypoxia-inducible factor 1-alpha
PI3K: Phosphoinositide 3-kinase
Akt: Protein Kinase B
VEGF: Vascular Endothelial Growth Factor
Gln: Glutamine
TCA: Tricarboxylic acid
DEGs: Differentially Expressed Genes
GO: Gene Ontology
KEGG: Kyoto Encyclopedia of Genes and Genomes
GSEA: Gene Set Enrichment Analysis
PPI: Protein-Protein Interaction
HUVECs: Human Umbilical Vein Endothelial Cells
LDBF: Laser Doppler Blood Flow
DUS: Doppler Ultrasound Signal
RT-PCR: Reverse Transcription Polymerase Chain Reaction
CCK8: Cell Counting Kit-8
mRNA: Messenger Ribonucleic Acid
cDNA: Complementary Deoxyribonucleic Acid
FPKM: Fragments Per Kilobase of transcript per Million mapped reads
H&E: Hematoxylin and Eosin
OD: Optical Density
PUs: Perfusion Units
Slc1a5: Solute Carrier Family 1 Member 5
Gls: Glutaminase
Glud1: Glutamate Dehydrogenase 1
BPTES: Bis-2-(5-phenylacetamido-1,2,4-thiadiazol-2-yl)ethyl sulfide
DM-α-KG: Dimethyl-α-Ketoglutarate

## References

[CR1] Bodineau C, Tomé M, Murdoch PDS, Durán RV. Glutamine, MTOR and autophagy: a multiconnection relationship. Autophagy. 2022;18(11):2749–50. 10.1080/15548627.2022.2062875. 35470752 10.1080/15548627.2022.2062875 PMC9629096

[CR2] Chang EI, Loh SA, Ceradini DJ, Chang EI, Lin S-e, Bastidas N, et al. Age decreases endothelial progenitor cell recruitment through decreases in hypoxia-inducible factor 1α stabilization during ischemia. Circulation. 2007;116(24):2818–29. 10.1161/CIRCULATIONAHA.107.715847. 18040029 10.1161/CIRCULATIONAHA.107.715847

[CR3] Eelen G, Dubois C, Cantelmo AR, Goveia J, Brüning U, DeRan M, et al. Role of glutamine synthetase in angiogenesis beyond glutamine synthesis. Nature. 2018;561(7721):63–9. 10.1038/s41586-018-0466-7. 30158707 10.1038/s41586-018-0466-7

[CR4] Fan W, Liu Z, Chen J, Liu S, Chen T, Li Z, et al. Effect of memantine on the survival of an ischemic random skin flap and the underlying mechanism. Biomed Pharmacother. 2021;143:112163. 10.1016/j.biopha.2021.112163. 34517281 10.1016/j.biopha.2021.112163

[CR5] García-Maceira P, Mateo J. Silibinin inhibits hypoxia-inducible factor-1alpha and mTOR/p70S6K/4E-BP1 signalling pathway in human cervical and hepatoma cancer cells: implications for anticancer therapy. Oncogene. 2009;28(3):313–24. 10.1038/onc.2008.398. 18978810 10.1038/onc.2008.398

[CR6] Ge J, Cui H, Xie N, Banerjee S, Guo S, Dubey S, et al. Glutaminolysis promotes collagen translation and stability via α-ketoglutarate–mediated mTOR activation and proline hydroxylation. Am J Respir Cell Mol Biol. 2018;58(3):378–90. 10.1165/rcmb.2017-0238OC. 29019707 10.1165/rcmb.2017-0238OC PMC5854958

[CR7] Hashimoto I, Abe Y, Ishida S, Kashiwagi K, Mineda K, Yamashita Y, et al. Development of skin flaps for reconstructive surgery: random pattern flap to perforator flap. J Med Invest. 2016;63(3.4):159–62. 10.2152/jmi.63.159. 27644551 10.2152/jmi.63.159

[CR8] Hensley CT, Wasti AT, DeBerardinis RJ. Glutamine and cancer: cell biology, physiology, and clinical opportunities. J Clin Invest. 2013;123(9):3678–84. 10.1172/JCI69600. 23999442 10.1172/JCI69600 PMC3754270

[CR9] Hou P, Kuo C-Y, Cheng C-T, Liou J-P, Ann DK, Chen Q. Intermediary metabolite precursor dimethyl-2-ketoglutarate stabilizes hypoxia-inducible factor-1α by inhibiting prolyl-4-hydroxylase PHD2. PLoS ONE. 2014;9(11):e113865. 10.1371/journal.pone.0113865. 25420025 10.1371/journal.pone.0113865 PMC4242664

[CR10] Huang H, Vandekeere S, Kalucka J, Bierhansl L, Zecchin A, Brüning U, et al. Role of glutamine and interlinked asparagine metabolism in vessel formation. EMBO J. 2017;36(16):2334–52. 10.15252/embj.201695518. 28659375 10.15252/embj.201695518 PMC5556263

[CR11] Jia S, Huang J, Lu W, Miao Y, Huang K, Shi C, et al. Global hotspots and future directions for drugs to improve the skin flap survival: a bibliometric and visualized review. J Pharm Anal. 2024;14(7):100948. 10.1016/j.jpha.2024.02.002. 39109384 10.1016/j.jpha.2024.02.002 PMC11300935

[CR12] Joyal J-S, Sun Y, Gantner ML, Shao Z, Evans LP, Saba N, et al. Retinal lipid and glucose metabolism dictates angiogenesis through the lipid sensor Ffar1. Nat Med. 2016;22(4):439–45. 10.1038/nm.4059. 26974308 10.1038/nm.4059 PMC4823176

[CR13] Kim B, Li J, Jang C, Arany Z. Glutamine fuels proliferation but not migration of endothelial cells. EMBO J. 2017;36(16):2321–33. 10.15252/embj.201796436. 28659379 10.15252/embj.201796436 PMC5556269

[CR14] Lee MS, Ahmad T, Lee J, Awada HK, Wang Y, Kim K, et al. Dual delivery of growth factors with coacervate-coated poly(lactic-co-glycolic acid) nanofiber improves neovascularization in a mouse skin flap model. Biomaterials. 2017;124:65–77. 10.1016/j.biomaterials.2017.01.036. 28188996 10.1016/j.biomaterials.2017.01.036

[CR15] Li M, Wang Y, Wei X, Cai W-F, Wu J, Zhu M, et al. AMPK targets PDZD8 to trigger carbon source shift from glucose to glutamine. Cell Res. 2024a;34(10):683–706. 10.1038/s41422-024-00985-6. 38898113 10.1038/s41422-024-00985-6 PMC11442470

[CR16] Li S, Zhao C, Shang G, Xie JL, Cui L, Zhang Q, et al. Α-ketoglutarate preconditioning extends the survival of engrafted adipose-derived mesenchymal stem cells to accelerate healing of burn wounds. Exp Cell Res. 2024b;439(1):114095. 10.1016/j.yexcr.2024.114095. 38759745 10.1016/j.yexcr.2024.114095

[CR17] Li Y, Liu Z, Yan H, Zhou T, Zheng L, Wen F, et al. *Polygonatum sibiricum* polysaccharide ameliorates skeletal muscle aging and mitochondrial dysfunction via PI3K/Akt/mTOR signaling pathway. Phytomedicine. 2025;136:156316. 10.1016/j.phymed.2024.156316. 39674120 10.1016/j.phymed.2024.156316

[CR18] Li Y, Jiang Q-l, Van Der Merwe L, Lou D-h, Lin C. Preclinical efficacy of stem cell therapy for skin flap: A systematic review and meta-analysis. Stem Cell Res Therapy. 2021;12(1):28. 10.1186/s13287-020-02103-w. 10.1186/s13287-020-02103-w PMC7791712 33413598

[CR19] Maxwell PH, Wiesener MS, Chang GW, Clifford SC, Vaux EC, Cockman ME, et al. The tumour suppressor protein VHL targets hypoxia-inducible factors for oxygen-dependent proteolysis. Nature. 1999;399(6733):271–5. 10.1038/20459. 10353251 10.1038/20459

[CR20] Ramakrishnan S, Anand V, Roy S. Vascular endothelial growth factor signaling in hypoxia and inflammation. J Neuroimmune Pharmacol. 2014;9(2):142–60. 10.1007/s11481-014-9531-7. 24610033 10.1007/s11481-014-9531-7 PMC4048289

[CR21] Rohlenova K, Veys K, Miranda-Santos I, De Bock K, Carmeliet P. Endothelial cell metabolism in health and disease. Trends Cell Biol. 2018;28(3):224–36. 10.1016/j.tcb.2017.10.010. 29153487 10.1016/j.tcb.2017.10.010

[CR22] Stephan D, Blatt S, Riedel J, Mohnke K, Ruemmler R, Ziebart A, et al. The impact of transfer-related ischemia on free flap metabolism and electrolyte homeostasis—a new in vivo experimental approach in pigs. J Clin Med. 2023;12(20):6625. 10.3390/jcm12206625. 37892763 10.3390/jcm12206625 PMC10607031

[CR23] Wang Z, Wang W, Shi H, Meng L, Jiang X, Pang S, et al. Gamma-glutamyltransferase of *Helicobacter pylori* alters the proliferation, migration, and pluripotency of mesenchymal stem cells by affecting metabolism and methylation status. J Microbiol. 2022;60(6):627–39. 10.1007/s12275-022-1575-4. 35437622 10.1007/s12275-022-1575-4

[CR24] Wu N, Yang M, Gaur U, Xu H, Yao Y, Li D. Alpha-ketoglutarate: physiological functions and applications. Biomol Ther. 2016;24(1):1–8. 10.4062/biomolther.2015.078. 10.4062/biomolther.2015.078 PMC4703346 26759695

[CR25] Wu H, Ding J, Li S, Lin J, Jiang R, Lin C, et al. Metformin promotes the survival of random-pattern skin flaps by inducing autophagy via the AMPK-mTOR-TFEB signaling pathway. Int J Biol Sci. 2019;15(2):325–40. 10.7150/ijbs.29009. 30745824 10.7150/ijbs.29009 PMC6367544

[CR26] Yang S, Hwang S, Kim M, Seo SB, Lee J-H, Jeong SM. Mitochondrial glutamine metabolism via GOT2 supports pancreatic cancer growth through senescence inhibition. Cell Death Dis. 2018;9(2):55. 10.1038/s41419-017-0089-1. 29352139 10.1038/s41419-017-0089-1 PMC5833441

[CR27] Yang R, Zhang G, Meng Z, Wang L, Li Y, Li H, et al. Glutamate dehydrogenase 1-catalytic glutaminolysis feedback activates EGFR/PI3K/AKT pathway and reprograms glioblastoma metabolism. Neuro Oncol. 2025;27(3):668–81. 10.1093/neuonc/noae222. 39446525 10.1093/neuonc/noae222 PMC11889723

[CR28] Zhang J-y, Zhou B, Sun R-y, Ai Y-l, Cheng K, Li F-n, et al. The metabolite α-KG induces GSDMC-dependent pyroptosis through death receptor 6-activated caspase-8. Cell Res. 2021;31(9):980–97. 10.1038/s41422-021-00506-9. 34012073 10.1038/s41422-021-00506-9 PMC8410789

[CR29] Zhong H, Chiles K, Feldser D, Laughner E, Hanrahan C, Georgescu MM, et al. Modulation of hypoxia-inducible factor 1alpha expression by the epidermal growth factor/phosphatidylinositol 3-kinase/PTEN/AKT/FRAP pathway in human prostate cancer cells: implications for tumor angiogenesis and therapeutics. Cancer Res. 2000;60(6):1541–5. 10749120

[CR30] Zhou K, Chen H, Lin J, Xu H, Wu H, Bao G, et al. FGF21 augments autophagy in random-pattern skin flaps via AMPK signaling pathways and improves tissue survival. Cell Death Dis. 2019;10(12):872. 10.1038/s41419-019-2105-0. 31740658 10.1038/s41419-019-2105-0 PMC6861244

