# Supplementary material for: Alpha-ketoglutarate promotes random-pattern skin flap survival by enhancing angiogenesis via PI3K/Akt/HIF-1α signaling pathway
Source: Cell Regen. 2025 Dec 22;14:54. doi: 10.1186/s13619-025-00264-8 (PMC12722632; doi:10.1186/s13619-025-00264-8)
Supplement: Supplementary file 1 — Supplementary Material 1. Table S1. Primers used in this study. [file 13619_2025_264_MOESM1_ESM.docx]

**Table S1.** Primers used in this study.

| Type | Gene | Forward primer | Revers primer |
| --- | --- | --- | --- |
| Mouse | Slc1a5 | 5'-TTGTGCTGCTGGATGGGAAG-3' | 5'-CGTAGGGAGGCTGAGATCCTG-3' |
|  | Gs | 5'-ATGCCTGCTGCTGGTGCA-3' | 5'-GGCTTGGTGGGACCTGTTG-3' |
|  | Gls1 | 5’-TGGCTGTGATGAGGGACTG-3’ | 5’-GAGGGGACCTGAGGAGGAT-3’ |
|  | Glud1 | 5'-TGGCTGTGATGAGGGACTG-3' | 5'-GAGGGGACCTGAGGAGGAT-3' |
|  | Ki67 | 5'-CGGAGTACCTGGACCTGCTG-3' | 5'-GCTGCTGGATGGCTGAGAGT-3' |
|  | CD31 | 5'-ACGCTGGCTTGCTACGACTG-3' | 5'-GTGGCTGTGGTGGTGGAACT-3' |
|  | HIF-1α | 5′-TCTCGGCGAAGCAAAGAGTC-3′ | 5′-AGCCATCTAGGGCTTTCAGATAA-3′ |
|  | Vegf | 5′-CTGCCGTCCGATTGAGACC-3′ | 5′-CCCCTCCTTGTACCACTGTC-3′ |
|  | IL-1β | 5′-TGCCACCTTTTGACAGTGATG-3′ | 5′-TGATGTGCTGCTGCGAGATT-3′ |
|  | IL-6 | 5′-AGCCAGAGTCCTTCAGAGAGAT-3′ | 5′-AGAGCATTGGAAATTGGGGT-3′ |
|  | iNOS | 5’-GACCAGAAACTGTCTCACCTG-3’ | 5’-CGAACATCGAACGTCTCACA-3’ |
|  | β-actin | 5′-GCCACTGTCGAGTCGCGT-3′ | 5′-GATACCTCTCTTGCTCTGGGC-3′ |
| Human | HIF-1α | 5’-ACAGCCTGGTGGAGATGTGT-3’ | 5’-GTGCTGGTCTTGGTTGTGAC-3’ |
|  | Vegf | 5’-TGCAGATTATGCGGATCAAACC-3’ | 5’-TGCATTCACATTTGTTGTGCTGTAG-3’ |
